# Lessons learned in implementing an Alcoholics Anonymous peer support model in Lilongwe, Malawi

**DOI:** 10.4102/jphia.v17i1.1343

**Published:** 2026-03-20

**Authors:** Katherine Boyd, Chifundo S. Tepeka, Thomas W. Psyata, Tommy Mthepheya, Thuy Bui, Caroline Kensler, Phillip Wagner

**Affiliations:** 1 Department of Internal Medicine, University of Pittsburgh Medical Center, Pittsburgh, United States of America; 2 Department of Internal Medicine, Kamuzu Central Hospital, Lilongwe, Malawi; 3 Department of Internal Medicine, Johns Hopkins School of Medicine, Baltimore, United States of America

## Introduction

According to the World Health Organization (WHO) STEPwise Approach to NCD Risk Factor Surveillance (STEPS) survey for risk factors for noncommunicable diseases (NCDs) conducted in Malawi in 2009, nearly 20% of men who are 25 years to 64 years of age in Malawi drink alcohol to excess. This survey utilised the WHO STEPwise approach in a nationwide cross-sectional study to elicit the impact of NCDs, gathering survey data on multiple risk factors, substance use and risk behaviours among them.^1^ A survey of University of Malawi students indicated that about 74% of male students and 27% of females reported episodes of heavy drinking.^2^ Alcohol use results in cirrhosis, cancer, and motor vehicle injuries, with a study at Kamuzu Central Hospital (KCH) indicating that about half of male pedestrians had used alcohol before the injury.^3^ Alcohol consumption also competes with essential expenditures on food, housing, and education, contributing to household poverty as research suggests that low-income Malawians spend disproportionately more on alcohol than their higher-income counterparts.^4^ Although Malawi’s overall alcohol per capita consumption is relatively lower than global averages,^5^ evidence of heavy episodic drinking, as in the aforementioned studies, suggests that the country may have a concentrated burden of harmful use.

Malawi has implemented only some of the WHO’s cost-effective interventions for the reduction of alcohol consumption at the population level. Established monitoring systems for alcohol use disorder and recommended policy disincentives are severely lacking in the public sector.^5,6^ Patients can be referred to mental health clinics for counselling, but limitations including inadequate clinic space, understaffing, and a lack of funding hamper optimal treatment for alcohol and substance use disorders.

Mutual support groups are composed of people in recovery, those seeking recovery, and those willing to help others who struggle with addiction. These programmes are not explicitly considered treatment or therapy. Twelve-step mutual support programmes (e.g. Alcoholics Anonymous or AA) represent readily available, no-cost community-based resources for individuals experiencing the harmful effects of alcohol. In Malawi, such programmes are not well known to the public and not readily available despite being accessible and prevalent in many other countries.^7^ However, as the central region has benefited from strong grassroots peer-led programmes to address NCDs, this model is ideal to support the treatment of alcoholism in a resource-limited setting.^8^

Studies have shown that patients treated in 12-step-oriented programmes demonstrated higher abstinence rates compared to patients treated in cognitive-behavioural-oriented programmes 1 year after treatment.^9^ Additional studies showed that early AA attendance predicts long-term sobriety apart from treatment.^10^ In a Cochrane analysis of pooled data from 27 earlier studies, researchers found a higher rate of abstinence among people who attended a 12-step programme with trained facilitators than those who participated in alternative therapies, and concluded that these programmes additionally likely produce significant relative cost savings.^11^ As these community support programmes are free, people can continue attending for years without worrying about insurance or cost.

## Programme goals and objectives

The goal was to establish peer-led self-help support groups for individuals suffering from the harmful effects of alcohol use in Malawi. The Community for Disease Prevention and Management (CDPM) is a grassroots Malawian organisation that has created and sustained peer support groups focusing on diabetes and hypertension management. They have partnered with the Mental Health Department at KCH to establish peer support groups for individuals suffering from harm related to alcohol. Supported by faculty from both the University of Pittsburgh School of Medicine and the Johns Hopkins School of Medicine, an interdisciplinary team of mental health providers, nurses, and physicians adapted the AA 12-Step programme for Malawians.

After the launch of this initiative, project team members and group leaders will plan to promote and encourage the growth of existing and the formation of new AA peer support groups in other communities. The objective of this study is to assess the feasibility of this intervention, providing insight into what challenges arise in AA implementation in this specific setting, elucidating how these challenges have been addressed, and serving as a guide for the formation of new groups in the region. Adjustment of the AA model to the Malawian context provides a framework for how a similar adaptation of a 12-step programme can be performed in other countries by working with community members to translate the materials, as well as utilising focus groups to understand barriers to implementation. By fostering international institutional partnerships to improve health for vulnerable citizens, it addresses two of the United Nations Sustainable Development Goals for Malawi.

## Methods and specifications

### Adapting the 12-step programme for communities in Malawi

**FIGURE 1:**
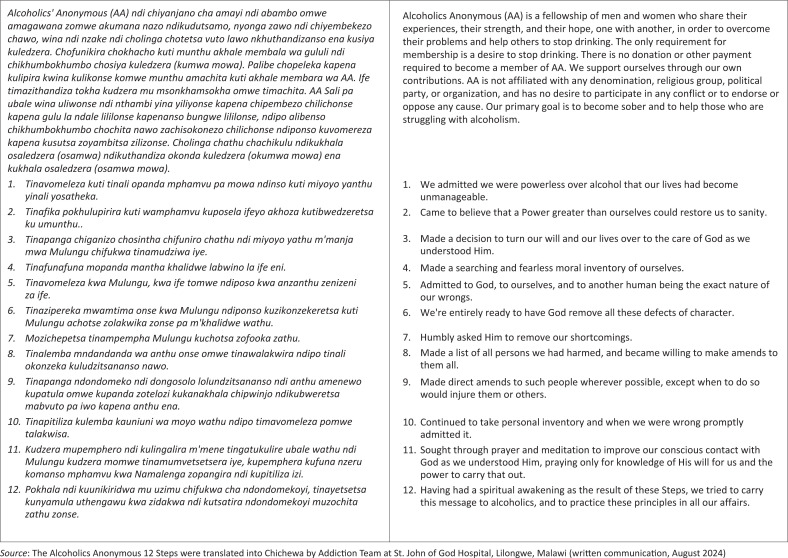
Twelve steps, Chichewa translation.

The CDPM partnered with the Mental Health Department at KCH to establish peer support groups for individuals suffering from harms related to alcohol. Support is provided by faculty from the University of Pittsburgh School of Medicine and Johns Hopkins School of Medicine. The collaborative team adapted the 12-Step programme for Malawians and trained group leaders to establish eight pilot groups in Lilongwe. The Alcoholics Anonymous *12 Steps* were translated from English into Chichewa by the Addiction Team at Saint John of God in Lilongwe (Figure 1).

### Training of supervisory team

Using the CDPM and KCH network, 12 interested individuals, including lay members from the existing CDPM peer support network and KCH clinical staff, were identified to serve as group leaders. They were given specific training on psychoeducation and the AA model by Malawian behavioural health experts with support from faculty from the two aforementioned United States (US) medical institutions.

**FIGURE 2:**
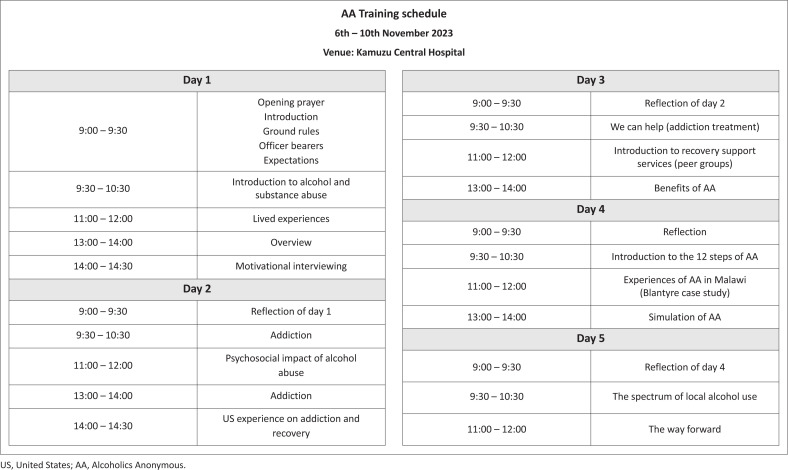
Initial group leader training schedule.

The initial training took place over 5 days at KCH. It involved multiple topics, including screening for hazardous alcohol use, psychoeducation, 12-step principles, spirituality in recovery, motivational interviewing, harm reduction, how to start and conduct meetings, and sponsorship (Figure 2). Throughout the pre- and post-training period, the supervisory team had meetings at frequent intervals with supporting faculty for project management guidance and continuing professional education on addiction and peer support.

### Formation of pilot groups

Those individuals who participated in the training committed to either starting a support group in their communities or supporting one or more of the pilot groups. Eight pilot groups were ultimately established in the semi-urban communities surrounding Lilongwe. The peer support group leaders documented such items as the number of participants, returning participants, attrition, demographics, group dynamics (cohesiveness, independence, expressiveness) and completion of AA steps.

### Supervisory visits

**FIGURE 3:**
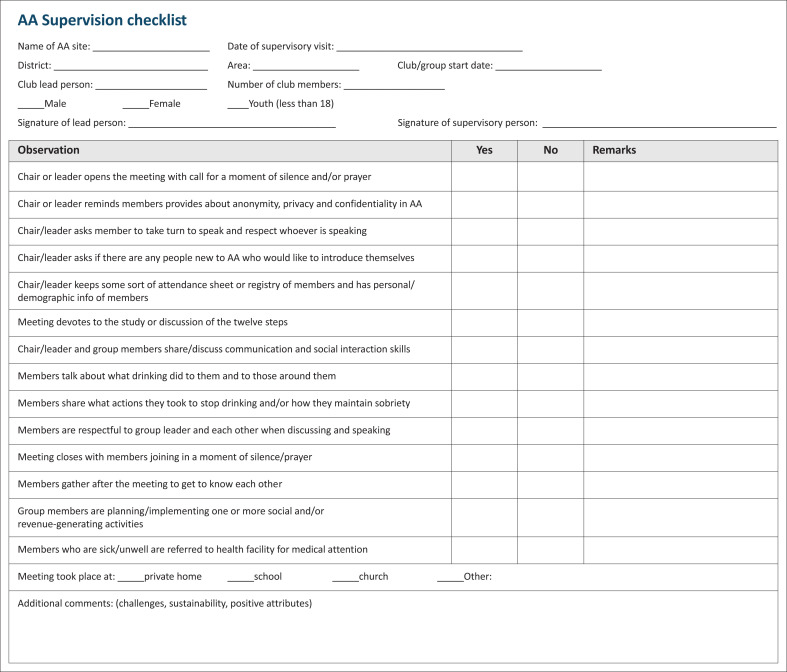
Supervisory checklist.

**FIGURE 4:**
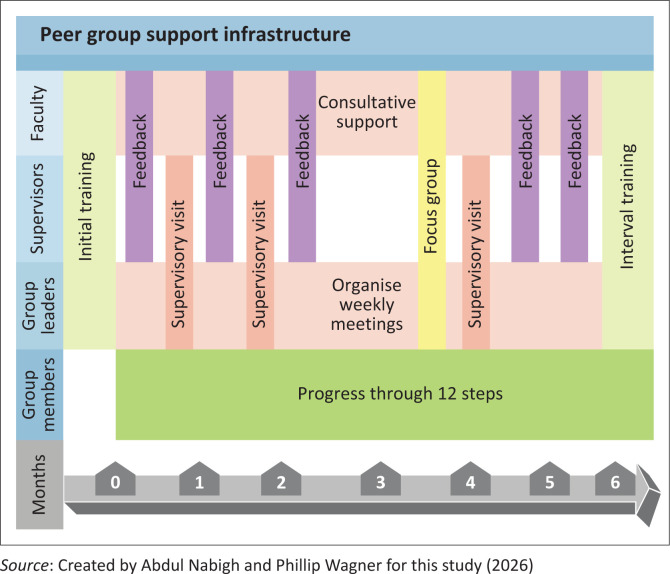
Multi-tiered peer support group structure by month.

Support was provided in the form of recruitment assistance, medical consultative support, and supervisory visits from project team members. Supervisory visits occurred during meetings, after which a two-way exchange of feedback was provided in the interest of continued improvement (Figure 3). Project team members used existing platforms such as ongoing diabetes and hypertension peer support group meetings, flyers and social media to promote this initiative. Three supervisory visits per group were conducted in the first 6 months. Meetings between on-the-ground supervisory project team members and supporting clinical faculty were held every 3 weeks (Figure 4).

### Focus groups with theme identification

Three months after the pilot groups began operating, consultative faculty led a focus group including project team members and group leaders. Information regarding programme scope, challenges in adoption and monitoring, and perceived efficacy was gathered. Structured discussion allowed each team member to reflect on the challenges, successes, and patterns they had witnessed during the course of supervisory visits. Challenges raised by participants at the focus group were then summarised and transcribed, and group discussion regarding next steps occurred in English. An inductive thematic approach was applied to the data to elicit themes. The focus group discussions were consolidated into themes related to weaknesses and challenges of the programme implementation. Training, education, and ongoing support were modified based on these perceived obstacles faced by the programme. No sensitive information was requested, discussed, or collected. Before the discussion, participants consented to not discuss specific individuals, groups, or locations. Formal ethics approval was not required as qualitative activities involved only members of the research and implementation team, with no data collection from external participants.

### Ethical considerations

This project was not research; no sensitive or personal data was requested or collected. While multiple authors have affiliations with academic institutions, this was a private project. As such, an ethical waiver was not requested. This article followed all ethical standards for research without direct contact with human or animal subjects.

## Results

Themes generated from inductive analysis were grouped into major and minor themes based on how large of an impediment the supervisory team perceived them to be.

A thematic analysis of implementing AA in Malawi reveals several primary obstacles to success. Participants often anticipated tangible rewards or incentives for attendance, influenced by prior foreign-led initiatives in the country, and struggled with the concepts of confidentiality. Material constraints, such as a lack of writing supplies and limited access to phone and online resources, further hindered engagement, while the stigma of alcohol use was compounded by mixed demographics created discomfort in group settings. In groups that were only several months old, a deficit of sponsorship limited support outside of group meetings.

**FIGURE 5:**
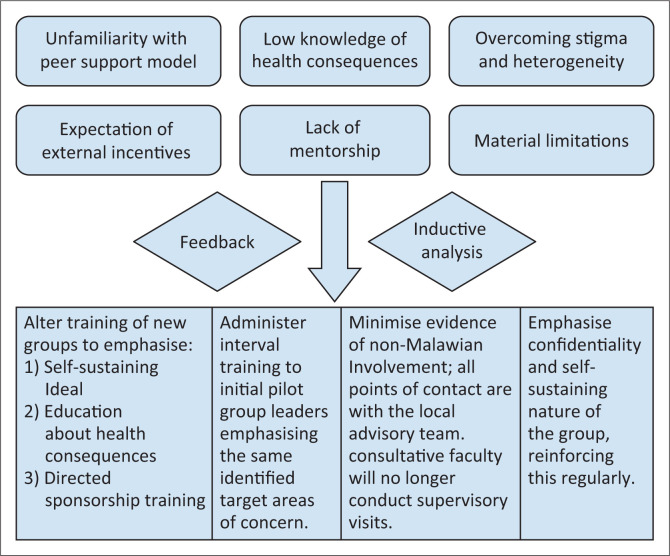
Adaptations based on thematic feedback.

The themes identified during the focus group served as the groupings into which further feedback was organised. To continually address challenges, a support structure composed of consultative support from medical faculty, a local supervisory team with experience in addiction, and group leaders trained in administering a 12-step programme regularly shared bidirectional feedback. Led by local team members, this initiative emphasises the self-sufficient nature of AA by consistently reinforcing peer support principles through observation and guidance. Feedback was used for adjustments in educational support and organisational restructuring (Figure 5).

### Major themes

#### Expectation of external incentives

*The groups want things to be done by the organisation. They expect that they will get money, T-shirts, or other items from attending the meetings. They are struggling with the self-sustaining nature of the clubs*.

Participants frequently requested tangible rewards such as T-shirts, food, or gifts in exchange for attendance or participation. Some participants requested preferential treatment from external parties including KCH, viewing their involvement in AA as grounds for additional or preferential clinical care. Responses also showed that some participants expected the organisation to provide structured replacement activities as part of the programme. Examples raised included employment opportunities or trade training. The presence of expatriates or foreign sponsors in meetings heightened these expectations, as community members associated foreign-led programmes with the administration of incentives. This dynamic also underscores a struggle with AA’s self-sustaining model, as it appears some participants initially viewed the programme as externally driven rather than internally motivated. These expectations of a transactional element to participation contrast with AA’s foundational principle of voluntary engagement.

#### Unfamiliarity with the peer support model

*One team member relayed a story: in one meeting, a group of people only attended to hear gossip about a specific neighbour. They laughed at her while she was sharing her story of addiction. She stopped sharing and stormed out of the meeting*.

*The group leaders are generally not talking about the importance of confidentiality and also not focusing on one specific step for that meeting. This has been ascribed to the newness of the programme and a lack of understanding. It has since improved with supervision, guidance, and practice*.

In the close-knit communities of target villages, having true anonymity was unlikely, making the need for confidentiality even more important. Maintaining confidentiality proved challenging; instances of participants breaking confidentiality were reported, likely because of a lack of awareness or respect for the need for privacy standards inherent in these arrangements. One unfortunate incident in a group’s first meeting involved community members attending in order to antagonise those individuals who were sharing their experience with alcohol misuse. In addition, confusion surrounding the structure of peer support meetings – such as the purpose of sharing personal experiences without direct advice or intervention – created misunderstandings among participants who may be more accustomed to directed forms of support.

#### Stigma of alcohol use and diverse demographics

*Some older men do not want to discuss their problems in front of women. Likewise, women are not comfortable around men. Because of this, men may not be honest about why their struggles in mixed groups*.

*It was noted that if men have family problems - like an issue with their wife - they do not want other women to know. They are often less likely to share. There are multiple requests to separate groups by age and gender*.

The theme of ‘Stigma’ emerged as a significant challenge in implementation, influencing both group dynamics and participants’ comfort levels. This was, of course, associated with the difficulties of anonymity in support groups meeting in small villages and the imperfection of confidentiality noted above. The stigma around alcoholism and recovery was the central factor, but this was exacerbated by the heterogeneous demographics of the groups, which included individuals of various ages and gender identities. This diversity made some participants feel uneasy, as discussing personal struggles with alcohol use in front of younger or older community members, or across gender identity lines, heightened discomfort and fear of judgement. Participants expressed a preference for separate, more homogenous groups, demarcated by age and gender identity, indicating that the cultural stigma around alcohol use was intensified by the community’s close-knit structure, where reputational concerns are particularly pronounced.

### Minor themes

#### Material limitations

Basic resources needed for the structure and processes of a peer support group are often lacking. Participants reported not having access to essential materials such as pens and paper, which limited their ability to write down or take notes on each of the 12 steps, personal reflections, or insights gained from meetings. This lack of materials hindered participants’ engagement with AA practices, as written exercises away from the group are a central component of the programme’s self-reflective journey. Furthermore, limited access to phones posed challenges for scheduling meetings, coordinating with group members, and delivering educational materials. In a context where technology is scarce, support groups face obstacles in maintaining regular communication and reinforcing programme teachings, which are often supported in higher-income countries by digital resources. These material limitations highlight the need for creative, low-tech solutions to support AA practices in resource-constrained settings and ensure that participants can engage fully in the programme despite these constraints.

#### A lack of standard mentorship

A miscellaneous category composed of related concerns, these challenges are linked by working within the confines of a developing novel and young infrastructure. Sponsorship – the relationship between a senior and junior group member – is a pillar of traditional 12-step groups. While progressing through the manualised steps is the primary intervention, with weekly meetings serving as both educational tools and fellowship, a sponsor serves as an external support and personalised guide between meetings. With young peer groups, there are no senior members able to mentor through experience. And while in traditional long-lived peer support groups, the group leader typically is someone who has themselves struggled with and overcome alcohol use in the past, the group leaders here were senior members of the community who are trained appropriately, but often have little or no personal experience with significant alcohol use.

#### A Lack of understanding regarding the health effects of alcohol

*People do not know alcohol use disorder is a medical problem here. However, there are a few people who want to stop the cycle they see friends and families going through with alcohol addiction*.

There seems to be little knowledge in the communities about the effects of drinking. The schools do not seem to teach about it, and it is not thought of as harmful in the culture.

Supervisory team members emphasised that harmful drinking is often interpreted within communities primarily through its visible social consequences rather than its health effects. Participants explained that individuals who drink heavily are commonly stigmatised because of their diminished ability to work, contribute financially, or fulfil social roles, yet the medical risks of alcohol use are rarely recognised or discussed. This informs not only why one of the pilot groups formed in a school (ages 11–15 years) but also chose to incorporate educational components aimed at providing foundational knowledge about the harms of alcohol use alongside the standard peer-support activities.

## Discussion

These findings echo broader challenges described in reviews of alcohol interventions in low- and middle-income countries (LMICs) where stigma, limited awareness of alcohol-related harms, and structural resource gaps commonly undermine implementation and uptake.^12^

There have been attempts to characterise and evaluate patient-level interventions targeting substance-use disorders in these settings, although the manuscripts available are not representative of the countries with the highest treatment gaps and the fewest resources, and therefore might not be transferable interventions. Staton et al.’s systematic review of patient-level alcohol use interventions in LMICs highlights that most evaluated approaches are brief, clinic-based interventions carried out by healthcare providers, with very few community-level, longitudinal interventions such as peer support groups.^13^ While the authors were targeting and studying interventions in LMICs, it is indicative of the limits of this approach that almost all the interventions they captured occurred only in middle-income countries. Similarly, a narrative and scoping review of community-based psychosocial substance-use disorder interventions and alcohol prevention in LMICs found similar barriers to engagement (a large treatment gap, context-specific stigma, limited health literacy, and material deficiencies) and also failed to include any interventions from low-income countries.^14^ Authors of these manuscripts called for studying feasibility of patient-level interventions in lower-income populations, evaluating interventions that are not led by healthcare professionals but trained lay people and characterised context-specific implementation issues.^13,14^

Satinsky et al., in a systematic review of peer-led interventions targeting substance use disorder, identified peer-delivered services as a promising strategy to expand access to substance-use care in LMICs. However, they also found that few studies describe how peer workers are trained, monitored to ensure adherence to their training, and supported sustainably by supervisory staff. Furthermore, the authors saw future opportunities for stakeholders from both high- and low-income countries to collaborate on peer-led interventions, combining specialised knowledge for the educational benefit of both parties.^15^

Our project details a pragmatic, multitier supervision model, combining regular supervisory visits, structured feedback loops, and iterative educational adjustments, that supports lay group leaders as they gain experience with the 12-step framework and incrementally achieve independence. This approach aligns with and operationalises recommendations calling for scalable, community-based psychosocial interventions that leverage non-specialist providers. Our data extend this literature by providing a detailed, country-specific account of how these forces manifest within a peer support framework in Malawi; transactional expectations for participation, discomfort discussing alcohol use across gender and age lines, and minimal baseline understanding of the health consequences of heavy drinking are context-specific barriers that have to be adapted to and planned for when targeted interventions are being implemented. Identifying these obstacles early in implementation has allowed for organisational adjustments and modified support for peer support trainers, and ongoing, targeted education and principal reinforcement for group leaders.

As the pilot groups stabilised, this supervisory structure enabled stepwise de-escalation of external oversight while simultaneously building local capacity. A second cohort of group leaders was trained using a refined curriculum that incorporated lessons from the initial thematic analysis, and new groups were launched in additional communities surrounding Lilongwe. By embedding AA-style groups within an existing grassroots peer-support network (CDPM) and progressively shifting responsibility to Malawian supervisors and leaders, our model illustrates a feasible pathway for transitioning from externally supported pilots to locally owned, community-driven recovery resources.

This experience and the barriers we encountered are telling. Our experience with the need for a school-based peer support group, the recurrent participant requests for age- and gender-segregated meetings, and the pervasive expectation of material incentives together highlight concrete design decisions future programmes must weigh while adapting AA or other peer support structures to similar settings. Even though resource constraints prevented us from creating fully stratified groups, explicitly acknowledging these cultural and logistical tensions and addressing them through enhanced orientation about the purpose, voluntary nature, and non-transactional ethos of AA allowed clearer communication to new members and group leaders. In sum, this project contributes novel, context-specific implementation data on how peer support interventions can be adapted, supported, and iteratively refined in a low-resource African setting, responding directly to calls for more detailed case studies of community-based alcohol interventions in sub-Saharan Africa.

## Conclusion and recommendations

Alcohol use disorder presents a persistent threat to physical and socioeconomic health, with a higher burden of this falling to lower-income individuals. The 12-step programme AA has long been a forum of structured community support that increases alcohol abstinence and subsequently may significantly reduce associated alcohol-related outcomes and medical care costs in the United States.^11^ The implementation of this model or similar models has not been attempted in many sub-Saharan nations, and further research is indicated to ascertain how best to locally adapt it for effectiveness in these cultural contexts.

This method of a multitiered supportive approach and frequent bidirectional feedback can be utilised in other areas of the world where alcohol use disorder presents an issue. The use of local supervisors and trainers aims to shift focus from external incentives to internal motivation, foster understanding of the peer support model, and build trust within culturally sensitive group dynamics. This localised, respectful approach strives to serve as a sustainable method to reinforce AA practice and support community-driven recovery in the long term in resource-limited regions.
